# Recording skin oxygenation by dual-wavelength ultra-wideband optoacoustic mesoscopy

**DOI:** 10.1038/s44303-026-00184-5

**Published:** 2026-07-10

**Authors:** Juan Aguirre, Andrei Berezhnoi, Benedikt Hindelang, Christine Gasteiger, Hailong He, Ulf Darsow, Tilo Biedermann, Vasilis Ntziachristos

**Affiliations:** 1 Chair of Biological Imaging, Central Institute for Translational Cancer Research (TranslaTUM), School of Medicine and Health & School of Computation, Information and Technology, Technical University of Munich, Munich, Germany; 2 Institute of Biological and Medical Imaging, Bioengineering Center, Helmholtz Zentrum München, Neuherberg, Germany; 3 Departamento de Tecnología Electrónica y de las Comunicaciones, Universidad Autonoma de Madrid, Madrid, Spain; 4 Instituto de Investigacion Sanitaria de la Fundacion Jimenez Diaz, Madrid, Spain; 5 Department of Dermatology and Allergology, Technical University of Munich, Munich, Germany; 6 DZHK (German Centre for Cardiovascular Research), partner site Munich Heart Alliance, Munich, Germany

**Keywords:** Diseases, Medical research, Optics and photonics

## Abstract

Microvascular oxygenation is a key physiological marker implicated in many health conditions, ranging from cardiovascular disease and diabetes to cancer, systemic inflammation, or sepsis, where microvascular-level dysfunction often precedes overt pathology. Ultra-wideband raster-scan optoacoustic mesoscopy (RSOM) offers unprecedented non-invasive visualization of tissue microvasculature, achieving three-dimensional (3D) resolution in the 10–30 micrometer range (axial, lateral). Nevertheless, the lack of availability of suitable multi-wavelength illumination sources has generally limited RSOM to single-wavelength studies of microvasculature morphology. Herein, we present a dual-wavelength ultra-wideband RSOM (DW RSOM) system for dynamic mapping of cutaneous microvascular oxygenation by integrating two diode-pumped solid-state lasers at 515 nm and 532 nm. The system was evaluated on healthy volunteers during post-occlusive reactive hyperemia (PORH) tests on the forearm and proximal nailfold (DRKS00037749 registered on 26th August 2025 on the German Clinical Trials Register). The system recorded 3D oxygenation changes, including ischemic decline, hyperemic overshoot, and recovery, consistent with invasive blood gas analyzer (BGA) measurements. Extracted parameters, such as oxygenation gradients and recovery times, correlated with expected vascular reactivity metrics. These findings validate DW RSOM as a portable prototype for the in vivo assessment of microvascular oxygenation in humans at a single microvessel level. By providing quantitative, layer-resolved imaging of skin microcirculation, DW RSOM offers a promising platform for extending single-wavelength RSOM to studying microvascular dysfunction and supports future clinical translation of optoacoustic technologies toward bedside vascular diagnostics.

## Introduction

Microvasculature oxygenation is emerging as a crucial biomarker in the pathophysiology of a wide range of diseases^1^. Microvascular perfusion and microvessel-level oxygen exchange are often the first tissue components to be compromised in cardiovascular disease (e.g., microangiopathy, ischemia), insulin resistance or diabetes (microvascular dysfunction), and cancer (tumor hypoxia, abnormal perfusion)^2–6^. Therefore, quantifying oxygen saturation (sO2) in the smallest vessels may provide early diagnostic and prognostic insight. For example, impaired microvascular oxygen delivery or increased heterogeneity of oxygenation can precede tissue damage and may correlate with outcomes in ischemic heart disease or diabetic complications^1^. Microvascular oxygenation metrics could complement conventional biomarkers (e.g., circulating markers, large-vessel imaging) by reflecting the local tissue-level physiology and pathophysiological burden more directly^7^.

Likewise, microvasculature oxygenation could be employed to detect the development of sepsis, a condition that affects 50 million people annually and leads to more than 11 million deaths^8^. Sepsis profoundly alters microvascular oxygenation dynamics^9,10^, making optoacoustic monitoring a promising tool for its early detection and prognosis. During sepsis, systemic inflammation, endothelial dysfunction, and microthrombosis lead to heterogeneous perfusion and impaired oxygen extraction at the microvessel level, even when macrocirculatory parameters such as blood pressure appear normal^9,10^. This “cytopathic hypoxia” results in spatially and temporally variable tissue oxygen saturation, which has been correlated with organ dysfunction and mortality^11^. Traditional monitoring methods, including pulse oximetry and mixed venous oxygen saturation, do not capture these localized microcirculatory disturbances that take place at the ten to hundreds micrometers scale. In contrast, optoacoustic imaging can non-invasively map hemoglobin oxygenation at microvascular resolution and in real time, providing insight into perfusion and oxygen utilization at the tissue level.

High-resolution performance is achieved in particular using ultra-wideband raster-scan optoacoustic mesoscopy (UWB-RSOM)^12^. This technique utilizes ultrasound bandwidths that may reach more than 100 MHz to resolve cutaneous microvasculature with close to 10–30 micrometer resolutions (axial, lateral)^13–15^. Recent work has addressed several limitations of earlier systems to deliver higher technology maturity that corrects for motion artifacts and allows fast acquisitions^16,17^. These advances have enabled consistent 3D vascular readouts (vessel density/diameter, branching, plexus delineation) and layer-resolved skin morphology. Clinically, UWB-RSOM has moved beyond feasibility into early translational studies across dermatology and systemic disease, such as detecting diabetes progression or cardiovascular disease (^18^, in press). Therefore, the technology is now positioned as a promising bedside tool for non-invasive microvascular assessment, with ongoing efforts focused on further miniaturization, increasing scan speed, standardization, and repeatability for longitudinal monitoring.

Despite this progress, the overwhelming majority of studies appearing in the literature typically utilize a single wavelength and resolve microvascular morphology. Multi-wavelength illumination using nanosecond pulses has been limited by the technology available today. In particular, spectral RSOM studies have been commonly performed with optical parametric oscillators (OPO), which allow wavelength tuning across the entire visible range and beyond. However, their high cost (above €100 K) and size (over 100 liters) limit their practicality for clinical applications. On the other hand, tunable Raman lasers have not delivered sufficient energy per pulse to result in meaningful RSOM implementations.

Diode-pumped solid-state (DPSS) lasers offer advantageous pulsing characteristics for optoacoustic mesoscopy. However, they are limited in the availability of wavelengths, as they are typically offered at discrete “harmonic” wavelengths associated with common gain media, e.g., neodymium-doped (Nd) crystals. The most common wavelength in the visible range is 532 nm, offered as the second harmonic (frequency doubling) of an Nd:YAG laser emitting at 1064 nm. However, a few implementations use a different gain medium, i.e., ytterbium-doped (Yd) crystals, with fundamental emission at 1030 nm and a second harmonic at 515 nm. In this study, we aimed to investigate whether the use of these two closely spaced DPSS lasers could be exploited as RSOM sources to reveal oxygenation changes in human skin microvasculature. For this reason, we developed a dual-wavelength (DW) 515/532 nm RSOM and applied it to a pilot human study under a post-occlusive reactive hyperemia (PORH) test. In the following sections, we present the system specifics and key findings of the study and discuss the potential applications of the dual-wavelength ultra-wideband RSOM (DW RSOM) system in clinical settings.

## Results

### The DW RSOM system and method

**Fig. 1:**
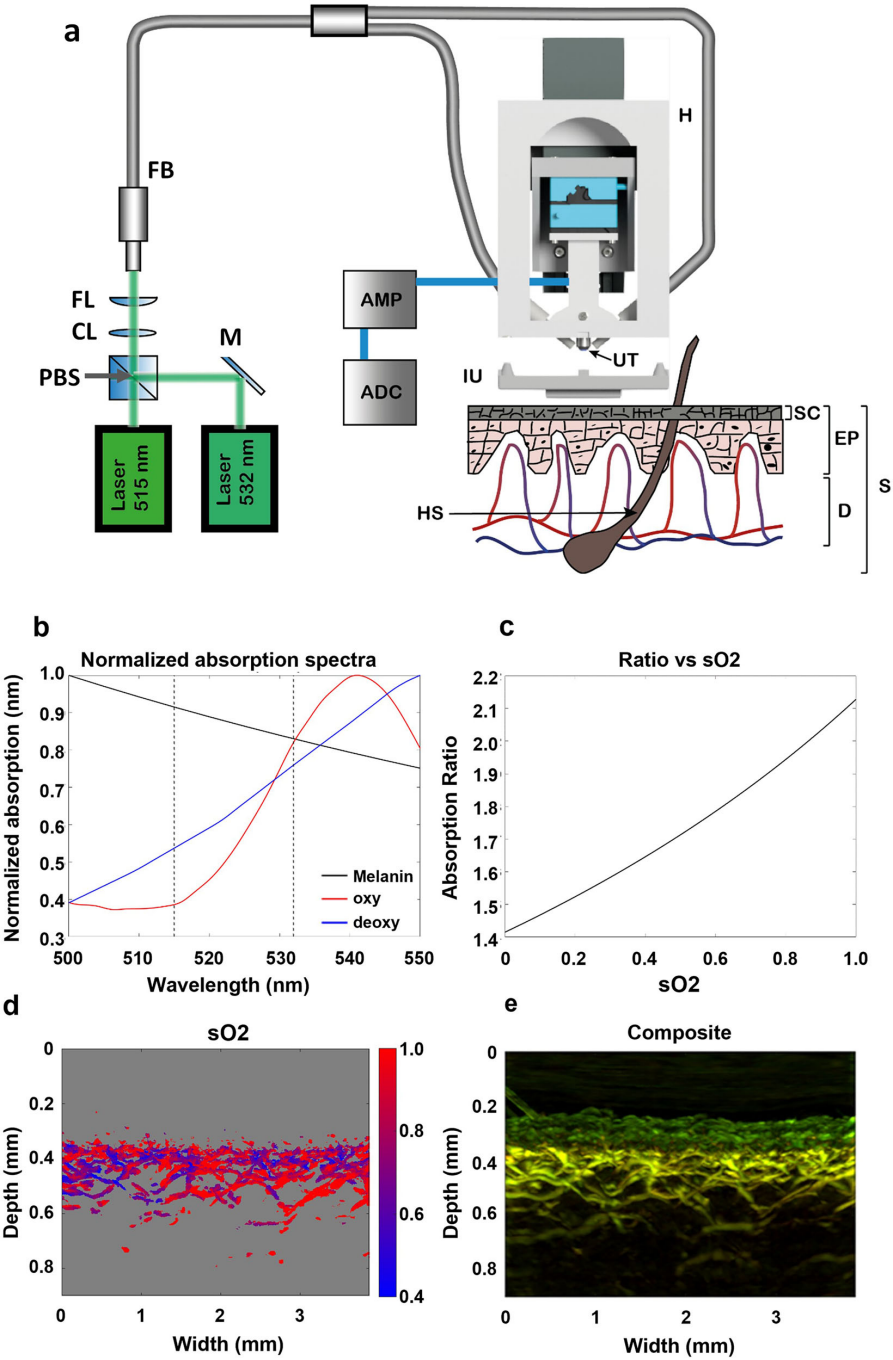
Dual-wavelength Raster-Scan Optoacoustic Mesoscopy (DW RSOM). **a** Schematic representation of the DW RSOM system and the experimental configuration. FB—fiber bundle, FL—focusing lens, CL—collimating lens, PBS—polarizing beam splitter, M—mirror, ADC—analog-to-digital converter, AMP—60 dBm amplifier, IU—detachable interface unit, UT—ultrasound transducer, H—DW RSOM holder, S—schematic of human skin, EP—epidermis, D—dermis, HS—hair shaft, SC—stratum corneum. **b** Absorption spectra for melanin, oxyhemoglobin (oxy), and deoxyhemoglobin (deoxy), (**c**) 515/532 nm absorption ratio in relation to oxygen saturation (s02), (**d**) Oxygen saturation map of skin microvasculature after thresholding the composite image (values below 0.9 representing venules and above representing arterioles) (**e**). The composite image shows the oxygenation gradient between arterioles and venules, with yellow indicating higher oxygenation and green indicating lower oxygenation. These values were used to segment arterioles (red) from venules (blue) in (**d**). The most superficial layer on (**e**) appears green due to melanin bias.

The DW RSOM (Fig.1a) system employed two pulsing DPSS lasers at 515 nm and 532 nm with a pulse duration of ∼1 ns. The two lasers were combined into a single optical path using a beam splitter and directed to a fiber bundle, which was split into two paths and placed in the RSOM scan head. Pulses from the two lasers were time-interleaved, achieving a 1 kHz combined pulse rate (0.5 kHz per laser). The combined pulse train was partially diverted via a beamsplitter to a photodiode, providing a trigger signal for an analog-to-digital converter (ADC). A single-element focused ultrasound transducer, with a broad bandwidth (∼10–120 MHz), was also attached to the RSOM scan head. The scan head was mounted onto motorized x, y stages for raster scanning over a 2D grid, with a z-stage to adjust focal distance.

During scanning, an A-scan (i.e., time series of optoacoustic signals) was recorded at each raster position. The step size between adjacent points was chosen to ensure adequate sampling of the lateral point spread function, as defined by the focal point of the ultrasound transducer (∼20 µm). The maximum scanning speed was limited by the laser repetition rate (1 kHz per wavelength). The collected sinogram (a stack of A-scans) was then beam-formed via 3D reconstruction methods (delay-and-sum or back-projection) to yield the volumetric image of absorption. To exploit the broadband nature of the data, the system performed frequency-band separation, i.e., separately reconstructed the low-frequency band (10-40 MHz) to resolve larger micro-vessels and structures, and the high-frequency band (40-120 MHz) to resolve smaller micro-vessels and structures. The two separate images with different colors (red for the low-frequency band and green for the high-frequency band) were merged to improve the co-localized rendering of both large and small structures.

The two wavelengths, 515 nm and 532 nm, employed were selected based solely on their technical availability, as explained in the introduction. Nevertheless, these wavelengths are positioned on opposite sides of the isosbestic point for oxygenated hemoglobin (HbO2) and deoxygenated hemoglobin (Hb) (Fig. 1b). Therefore, their use could, in principle, be employed to differentiate between these two hemoglobin forms. Figure 1b also plots the spectrum of melanin to indicate the potential slight bias in computing HbO2 and Hb when only two wavelengths are employed. This bias arises since melanin absorption monotonically declines with increasing wavelength, showing ∼10% less normalized absorption at 532 nm compared to 515 nm. In other words, for a two-wavelength system, it is advantageous for the two wavelengths to be closely spaced. Nevertheless, in contrast to hemoglobin, which is distributed throughout the sampled volume, melanin is generally constrained in the stratum basale, a skin layer only 10–20 micrometers thick; therefore, it may act as a neutral density filter.

Figure1c plots the ratio of intensities collected at 532 nm over 515 nm, as a function of oxygen saturation (see methods, Eq. 4). The graph serves to demonstrate that at 100% oxygen saturation, the ratio of absorption coefficients at 532 nm over 515 nm is ∼2.1 and gradually drops to ∼1.4 for fully deoxygenated hemoglobin (0% saturation). We use the ratio from Fig. 1c to separate arterioles from venules (Fig.1d) and estimate oxygen saturation maps of the skin microvasculature (Fig.1e). Generally, images acquired at two wavelengths are plotted herein as composite images (Fig.1e), using green color for the image acquired at 515 nm and red color for the image acquired at 532 nm. When the green and red images are superimposed, yellow-orange colors indicate higher oxygenation, while green colors indicate lower oxygenation. This oxygenation color map is possible because the absorption of deoxygenated hemoglobin is stronger at 515 nm, while oxygenated hemoglobin exhibits stronger absorption at 532 nm. Due to the higher absorption of melanin at 515 nm than at 532 nm, signals from melanin-rich layers appear in green. Segmentation of the vessels using this color difference (Fig.1d) can be used to separate arterioles (red) from venules (blue) by differentiating fully oxygenated from partially oxygenated microvessels.

### Clinical studies

**Fig. 2:**
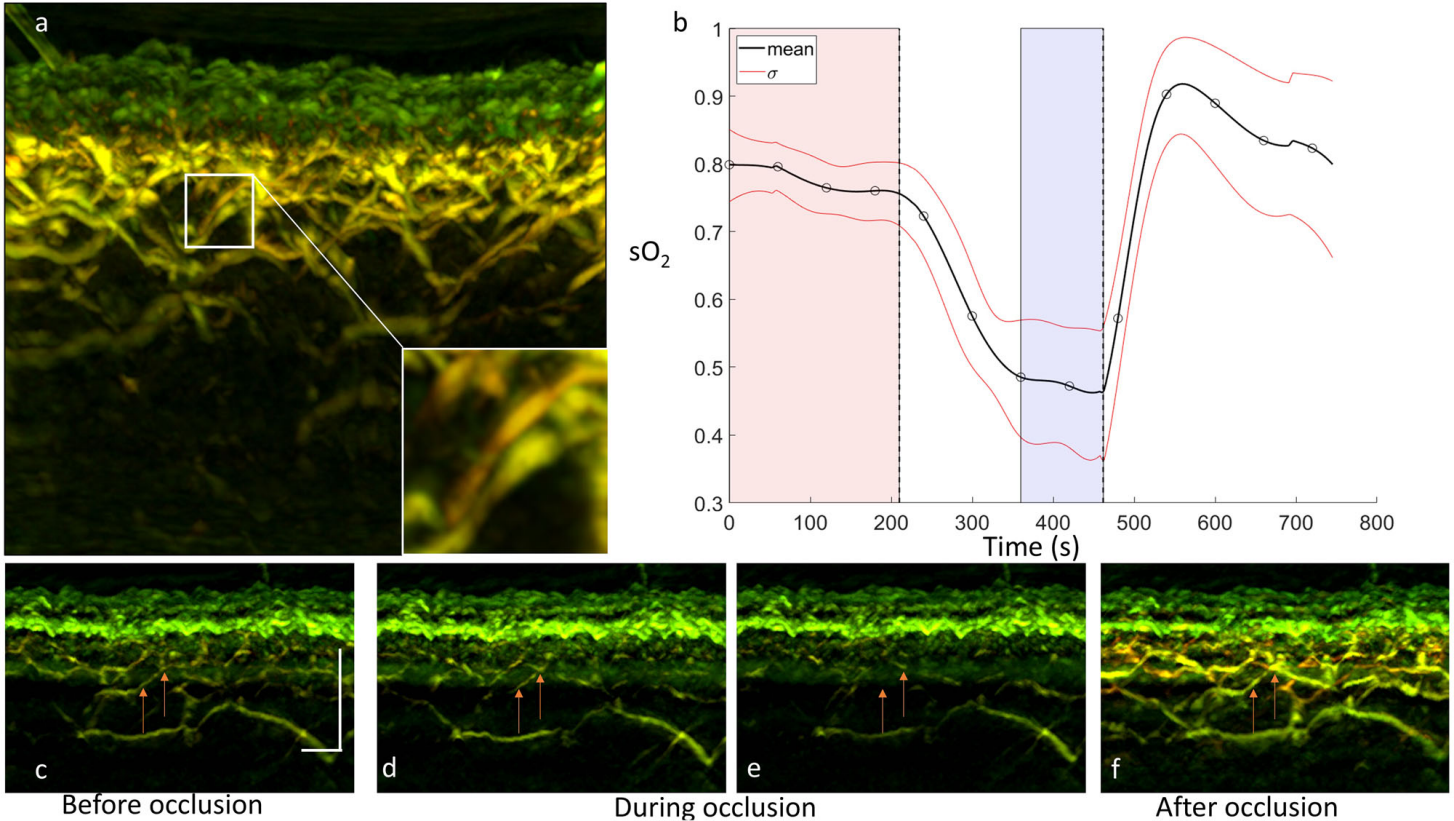
Oxygenation changes in dermal microvasculature of the forearm during Post-Occlusive Reactive Hyperemia (PORH) observed with a Dual-wavelength Raster-Scan Optoacoustic Mesoscopy (DW RSOM) system. **a** Merged maximum intensity projections (MIPs) of skin acquired at 515 nm (green color) and 532 nm (red color); the inset is a magnified view showing the oxygenation difference between vessels. **b** Oxygen saturation (sO2) measured with DW RSOM (s02-DW RSOM) before (0-205 s), during (206-460 s), and after (460-750 s) systolic occlusion. The vertical dashed lines mark the application and then release of the cuff. A blood gas analyzer (BGA), used to benchmark oxygen saturation values at the baseline (red rectangle) and ischemic plateau (blue rectangle), returned values of 87.5 ± 2.5% and 50 ± 10%, respectively. **c**-**f** MIP of skin (4 images: 1 before, 2 during, and 1 after the occlusion). The arrows indicate vessels that vanish and reappear following the occlusion and release of the cuff. Scale bars represent 500 µm.

In a clinical study (approved by the TUM Ethics Committee, DRKS00037749 on the German Clinical Trials Register), we applied the system to 11 human volunteers during a PORH test. A summary of the results obtained during vascular occlusion test (VOT) imaging sessions on the forearm is presented in Fig. 2. Figure 2a illustrates a cross-sectional RSOM image of human forearm skin imaged using DW RSOM. The images represent maximum intensity projections (MIP) of 3D reconstructed data sets acquired at both 515 nm and 532 nm, depicted in composite form, i.e., projecting the 515 nm image in green and the 532 nm image in red, as described above. Figure 2b plots the average oxygenation changes measured by the DW RSOM system in all 6 healthy volunteers. The oxygenation values are represented by the black line, while the red lines represent the standard deviation observed in all measurements. Two dashed vertical lines mark the occlusion (200 s) by the cuff and its release (470 s). The sO2 values before the occlusion obtained using DW RSOM (sO2-DW RSOM) were stable at 80 ± 10% (Fig. 2b, red region). The occlusion was followed by a gradual decrease in blood oxygenation in the forearm microvasculature for 150 s until an ischemic plateau was reached (Fig. 2b, blue region). The approximate sO2-DW RSOM value computed during this plateau, using the ratios in Fig. 1c, was 46 ± 9%. For comparison, sO2 values were also measured with a blood gas analyzer (sO2-BGA), where the blood was taken from the dermal layer in close proximity to the imaged region. The sO2-BGA values before the occlusion were in the range of 87.5 ± 2.5%, while the sO2-BGA values during the occlusion were in the range of 50 ± 10%, with both values lying within the margin of error associated with the simple ratiometric computation followed in this study. Considering the number of healthy volunteers *n* = 6, imaging-based values of 80 ± 10%, and the blood-gas values of 87.5 ± 2.5% of sO2 paired Student’s *t* test gives *p* value of 0.097, which indicates that the difference between the two methods is not statistically significant (*p* > 0.05).

The prominent oxygenation peak (Fig. 2b, 560 s) after release of the cuff indicates a hyperemic response and an increase in the microvasculature oxygenation. The sO2-DW RSOM value peaks at 92 ± 8%, and values are transiently above the baseline average measured prior to occlusion before they revert to the expected values of 80 ± 12% within approximately 200 s. The ischemia downslope was found to be 14 ± 6%/min and was obtained by plotting the linear decrease in the sO2-DW RSOM from the time of occlusion until the start of the ischemic plateau. The time to recovery, i.e., the time taken for sO2-DW RSOM to revert to the initial mean after the release of the cuff, was found to be 38 ± 6 s. The rate of increase in oxygen saturation after cuff release was 41 ± 12%/min.

**Fig. 3:**
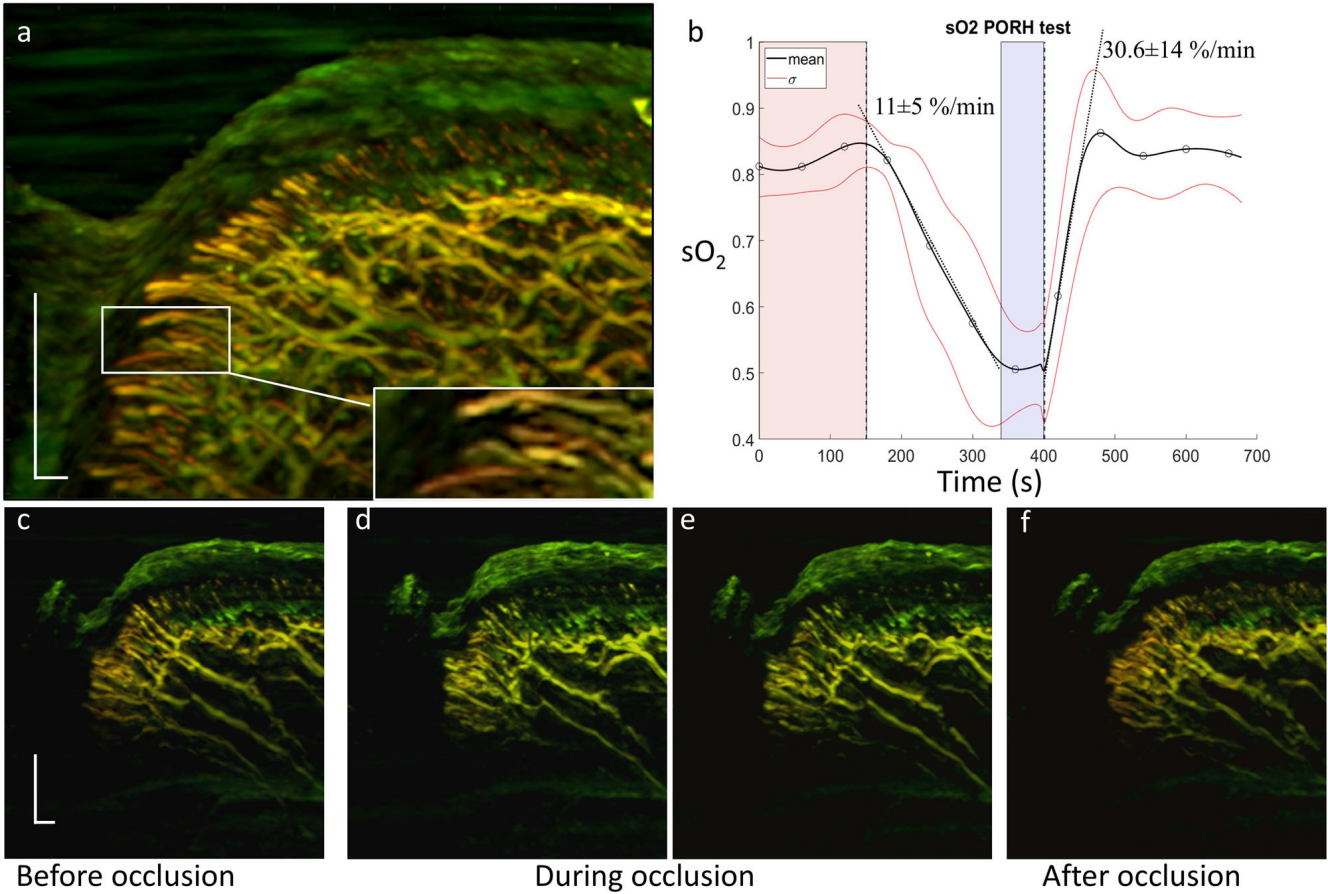
Oxygenation changes in the proximal nailfold microvasculature during a Vascular Occlusion Test (VOT) observed with a Dual-wavelength Raster-Scan Optoacoustic Mesoscopy (DW RSOM) system. **a** Merged maximum intensity projections (MIPs) of the nailfold microvasculature acquired at 515 nm (green) and 532 nm (red); the inset is a magnified view showing the oxygenation difference between capillary loops. **b** Oxygenation saturation measured with a DW RSOM (sO2-DW RSOM) before (0–150 s), during (151–400 s), and after (400–700 s) systolic occlusion. The vertical dashed lines mark the application and then release of the cuff. A blood gas analyzer (BGA) used to benchmark oxygen saturation values at the baseline (red rectangle) and ischemic plateau (blue rectangle) returned values of 90 ± 5% and 50 ± 10%, respectively. Dashed lines indicate the ischemia downslope attributed to the systolic occlusion (left dashed line) and the oxygen saturation upslope corresponding to the hyperemic reaction (right dashed line), and their respective values are displayed above the lines. **c**–**f** MIP of the proximal nailfold (4 images: 1 before, 2 during, and 1 after the occlusion). Scale bars represent 250 µm.

Figure 2c-f show a representative time sequence of DW RSOM images obtained for a representative measurement. The oxygenation changes recorded by DW RSOM in the forearm microvasculature before, during, and after occlusion by an arm cuff are taken ∼2 min apart. Figure 2c depicts the oxygenation state before the systolic cuff occlusion. Figure 2d,e illustrate the decrease in the oxygenation and overall blood volume of the dermal microvasculature during occlusion. The decrease in blood oxygenation is reflected by a shift in image colors toward more green tones from panel 2 d to 2e. After the cuff is released, the microvessels become redder (Fig. 2f), indicating an overshoot in blood oxygenation. Furthermore, the release of the cuff is also accompanied by the reappearance of vessels and the emergence of previously indiscernible vessels (marked with orange arrows in Fig. 3f) due to hyperemia.

We repeated the PORH test in 5 healthy volunteers, this time positioning the DW RSOM system to image the microvasculature in a human proximal nailfold. Figure 3a shows a representative image of the nailfold microvasculature, demonstrating fine details of the underlying capillary loops. The capillary loops are oriented horizontally at the proximal nailfold, and they rise up as they move away from the nailfold (to the right in Fig. 3a). As in Fig. 2, the orange-brown shade represents arterioles, which contain higher concentrations of oxygenated hemoglobin, and the yellow shade represents venules with increased concentrations of deoxygenated hemoglobin. The inset in Fig. 3a shows an enlarged section of the capillary loops from the field of view indicated with a solid white box on the main image. Figure 3b shows the quantitative results of the oxygenation change in the proximal nailfold as a result of the systolic occlusion applied to the forearm. The black line shows the mean sO2-DW RSOM values calculated from measurements in the 5 volunteers, and the red lines depict the standard deviation. Two dashed vertical lines mark the beginning (150 s) and end (400 s) of the occlusion. The red- and blue-shaded areas indicate the baseline before occlusion and the lowest sO2 values measured during occlusion, respectively. The average sO2 value before the occlusion was 87 ± 2%, which was consistent with the laboratory sO2-BGA values (90 ± 5%). Similarly to the findings in the forearm, the systolic occlusion was followed by a gradual decrease in microvasculature oxygenation in the nailfold for around 180 s, plateauing at an sO2 value of 58 ± 8%, which is in good agreement with the sO2-BGA values measured in the fingertip (50 ± 10%). The release of the cuff was followed by a hyperemic response and a concomitant increase in sO2 to a maximum of 88 ± 7% (maximal hyperemic response). After reaching this peak value, sO2 decreased moderately to 85 ± 5%. The ischemia downslope attributed to the systolic occlusion and measured in the proximal nailfold was found to be 11 ± 5%/min. The microvasculature oxygenation in the proximal nailfold quickly recovered after release of the occlusion, exhibiting a less prominent peak compared to the forearm values, with a calculated time of recovery of 68 ± 22 s. The oxygen saturation upslope corresponding to the hyperemic reaction was calculated to be 31 ± 14%/min.

## Discussion

We developed a portable DW RSOM system and investigated whether the closely spaced 515 nm and 532 nm wavelengths, imposed by the technical specifications of available lasers, could effectively resolve oxygenation changes in humans. To perform this investigation, results from the DW RSOM system were compared with blood measurements obtained invasively and analyzed using a laboratory BGA. The DW RSOM revealed oxygenation changes in response to the application and release of systolic occlusion in 11 healthy volunteers, with measurements taken from within the forearm or the nailfold. These findings were consistent with control measurements from the BGA, and the tissue oxygenation responses recorded generally matched with reported values during occlusion tests assessed using NIRS^19^. Nevertheless, the RSOM method employed herein is not affected by light scattering and photon diffusion. Therefore, it achieves higher detail and precision in quantifying oxygenation at a single microvessel level.

The DW RSOM weighs around 5 kg, and therefore it can be made portable using a cart, or used as a benchtop system. It renders 3D absorption maps faster and with higher resolution than previously reported multispectral optoacoustic skin imaging systems^20–22^. The higher resolution is achieved by incorporating a high-frequency ultra-broad bandwidth ultrasound transducer and Q-switched DPSS lasers. The laser pulse width, close to 1 ns, allows the generation of broadband high-frequency optoacoustic signals, while the transducer’s frequency range of 10–120 MHz facilitates rendering of structures at 10 µm in axial resolution. The lateral resolution, defined by the focusing ability of the ultrasound detector, is ∼30 µm. The high repetition rates of the employed lasers, operating at 500 pulses per second, afford imaging speeds at least an order of magnitude faster than systems using optical parametric oscillators for wavelength switching, which typically operate with 20-50 pulses per second.

3D oxygenation maps acquired during the occlusion test by DW RSOM yielded several clinically relevant parameters, including the oxygenation downslope (indicating ischemia), minimum sO2, oxygenation upslope of the hyperemic response, time to recovery, and maximal hyperemic response. Photoacoustic microscopy has been used previously to record oxygenation changes in response to occlusion. However, the speed and resolution of the system used were insufficient to render 3D changes in microvasculature oxygenation and allow the calculation of the VOT parameters^20^. The ischemia downslope is linked to metabolic activity and the rate of oxygen consumption from the microvasculature by the surrounding tissue. Oxygenation upslope, time of recovery, and maximal hyperemic response reflect reperfusion speed and the removal of deoxygenated blood, which correlate with endothelial function^23^. The calculated rate of oxygenation downslope, 14 ± 6%/min, exceeds previously reported values of sO2 using NIRS in the forearm, but is comparable to the values extracted from the thenar^19^. The changes in sO2-DW RSOM in the proximal nailfold microvasculature during VOT in response to arterial occlusion were recorded for the first time, to the best of our knowledge, at the high depth to resolution ratios characteristic of RSOM. Multispectral capillaroscopy cannot match the penetration depth^24^. The upslope of the sO2 curve during the hyperemic response is lower than reported previously using NIRS^19^. This discrepancy is possibly due to the lack of precision in NIRS caused by photon scattering^25^, as indicated by several reports discussing the contribution of the signal from skin to NIRS measurements^19^. To summarize, metabolic activity can vary for muscle and skin tissue, which alters oxygen consumption and other VOT parameters.

A caveat of in vivo skin oxygenation measurements is the interference of the optoacoustic signal from blood by melanin. Many segmentation approaches are based on spectral unmixing for melanin segmentation and require at least 3 wavelengths to separate melanin, oxyhemoglobin, and deoxyhemoglobin^26^. Using 3 wavelengths leads to increased acquisition time, higher system costs, and hardware complexity. In this work, we show that using only 2 wavelengths and a thresholding method effectively separates blood and melanin signals. Such an approach led to a decrease in error in calculating oxygenation and VOT parameters. Thus, this approach has the potential to facilitate measurements in skin with stronger pigmentation even when utilizing only 2 wavelengths. Furthermore, this study shows that our 2-wavelength approach can potentially be employed by other optoacoustic techniques seeking to provide oxygenation measurements of the skin microvasculature. Importantly, future work should address the possible effects of the melanin layer on the accuracy of the S02 measurements. We foresee that a simple correction factor may be sufficient to correct for the melanin contribution due to the short distance between wavelengths and the fact that the melanin layer is highly localized in the outer layers of the skin. Overall, the color separation of microvessels can effectively enable their separation into arterioles and venules. In the future, segmentation taking into account both the total hemoglobin content and the spectral separation may improve the morphological identification and functional characterization of the microvasculature.

The DW RSOM system has shown great potential for monitoring oxygenation changes in human cutaneous microvasculature. However, in contrast to multi-channel optoacoustic implementations at lower frequencies, DW RSOM systems operate in real-time^27,28^. In the future, the acquisition speed of the RSOM system (1 image/minute) should be further improved to better capture rapid oxygenation changes during the hyperemic response. As of today, the fast variations represented in the S02 temporal dynamics of Figs. 2b and 3b must be taken with caution since the acquisition speed of the system is slightly below one minute. Further in-depth studies of the effects of light fluence on oxygenation measurements are also needed to achieve quantitative measurements of oxygenated and deoxygenated hemoglobin concentrations, including elevated oxygenation in nailfold microvessels. Moreover, SO2 trends at the single microvessel level should also be studied. Furthermore, in analogy to advances in fluorescence imaging^29,30^, RSOM would benefit from the development of tissue-simulating phantoms that can ensure quality control and standardization in clinical applications of the technique. Quality control is particularly important for DW RSOM, since intensity changes in the light sources used can lead to a bias of the ratio employed to calculate the sO2 values, yielding systematic errors if left unchecked.

In summary, dual-wavelength operation at two closely matched wavelengths (515 nm and 532 nm) was shown to be effective in dynamically resolving oxygenation changes associated with cutaneous microvasculature in response to VOT in humans. The acquisition speed and temporal resolution of the DW RSOM system were improved by implementing lasers with faster repetition rates and narrower pulse widths, which greatly facilitated the calculation of clinically relevant VOT parameters. Improved spatial resolution allowed monitoring of oxygenation changes within the smallest vessels in the human body with high spatial specificity. Furthermore, the current portability of the system facilitates implementation and translation into clinical trials, which could enable studies or even early diagnosis of the wide range of diseases, including autoimmune diseases, associated with microvascular dysfunction through non-invasive testing of microvasculature function, oxygenation, and its alterations. Due to the absence of a real gold standard for microvessel oxygenation measurements, RSOM can become the key modality for detailed oxygenation studies of the superficial microvasculature. Nevertheless, speed improvements and quality control, through appropriate standards, can ease the dissemination of the modality into routine clinical settings.

## Methods

### DW RSOM system

In order to assess oxygenation changes in the skin microvasculature, we upgraded a previously developed in-house UWB-RSOM system^15^. The main components of this system are shown in Fig. 1a. Two pulsed single-wavelength lasers (Wedge HB.532, Bright Solutions, Pavia, Italy; Flare NX 515-0.6-2, Coherent, Germany) generated the optoacoustic signals. The pulse widths of both lasers did not exceed 1.2 ns, while the repetition rate could be adjusted from 17 Hz to 2500 Hz for the 515 nm laser and from 1 Hz to 10 kHz for the 532 nm laser. The lasers were coupled into custom-designed fiber bundles (CeramOptec, Riga, Latvia). The maximum energy deposited on the skin surface did not exceed safe exposure limits^31^. A customized piezoelectric transducer with a 55 MHz central frequency and a 110% relative bandwidth (55 MHz, Sonaxis, Besancon, France) detected broadband optoacoustic signals generated in the skin. The fiber bundles and the transducer were raster-scanned over the field of interest using precise piezoelectric stages (Physik Instrumente, Karlsruhe, Germany). The illumination spot was aligned with the focal point of the transducer (3 mm distance from the transducer). The detected signals were amplified with a low-noise amplifier (63 dB; AU-1291, MITEQ, Hauppauge, New York, USA) and digitized by the high-speed DAQ at a 1 GS/s sampling rate (EON-121-G20; Gage Applied Technologies, Montreal, Canada). A compact 3D-printed scanning head encapsulated the fiber bundle, transducer, and piezoelectric stages. In order to ensure acoustic coupling of the optoacoustic signal, the transducer and fiber bundles were immersed in water within a small compartment of the interface unit (Fig. 1, IU). The interface unit was attached to the skin surface using double-sided tape, and a thin transparent foil was used to avoid water leakage.

### Imaging protocols and image formation

The images were collected in a clinical study (approved by the TUM Ethics Committee and registered on 26^th^ August 2025 with the German Clinical Trials Register - DRKS00037749) in accordance with the Declaration of Helsinki. We followed two imaging protocols: one for imaging the forearm and one for imaging the nailfold. All participants signed an informed consent form before voluntary inclusion in the study.

Six volunteers were imaged using the forearm protocol. The optoacoustic signals were acquired over a 4×1 mm^2^ field of view (FOV) on the skin of the forearm. The acquisition time of each scan did not exceed 1 min. The acquisition time includes the time required to launch the acquisition, make the scan and store the data; therefore, it has certain variability. The lasers generated light pulses in an interleaved manner such that the acquired optoacoustic signals for each wavelength were essentially co-registered. The optoacoustic signals were acquired for approximately 200 s prior to application of the occlusion (i.e., 3-4 acquisitions before applying the cuff). Once the cuff was applied, we acquired four data sets before releasing the cuff (470 s after applying the cuff). 5 more data sets were acquired after cuff release. The entire imaging session lasted 750 s. Prior to reconstruction of the recorded signals, the acquired data sets were corrected for motion artifacts and separated for each wavelength, so that signals for each wavelength could be reconstructed separately^16,32^. For reconstruction, we used the so-called universal back-projection algorithm.

A very similar acquisition protocol was followed for assessing oxygenation changes in the proximal nailfold of the finger. In this case, 5 volunteers were imaged. The only difference in the imaging protocol was that the acquisition time before the occlusion was limited to 150 s. The cuff was released at 400 s, and the imaging was finished at 700 s.

For both the nailfold and the forearm measurements, the maximum penetration depth achievable by RSOM using 515 nm and 532 nm is 1.5 mm.

### Calculation of oxygen saturation using DW RSOM

The RSOM reconstructed images obtained at the two wavelengths were processed to derive the sO2 values in each volume element of the images. Then, a segmentation algorithm was applied to discriminate the voxels corresponding to the microvascular structure from those corresponding to melanin and noise.

The sO2 values were calculated as:

1 $${sO}2=\,\frac{{C}_{{oxy}}}{{C}_{{oxy}}+{C}_{{deoxy}}}$$

whereby ${C}_{{oxy}}$ and ${C}_{{deoxy}}$ are the concentrations of oxyhemoglobin and deoxyhemoglobin in the blood. SO2 and oxygen saturation are terms that can be interchanged, while oxygenation is a more qualitative and global term referring to “amount of oxygen” under which the particular term SO2 is included.

Assuming that oxyhemoglobin and deoxyhemoglobin are the chromophores that generate the optoacoustic signal, the initial pressure (${p}_{0}\left(\vec{r},\lambda \right)$), which corresponds to the intensity values of the reconstructed voxels, can be expressed as follows:

2 $${p}_{0}\left(\vec{r},\lambda \right)=\,\Gamma \phi \left(\vec{r,}{\mu }_{a},{\mu }_{s},g,\lambda \right)({\varepsilon }_{{oxy}}{\left(\lambda \right)C}_{{oxy}}\left(\vec{r}\right)+{\varepsilon }_{{deoxy}}\left(\lambda \right){C}_{{deoxy}}\left(\vec{r}\right))$$

where Γ is the Grüneisen parameter, and ϕ is light fluence; ${\varepsilon }_{{oxy}}$ and ${\varepsilon }_{{deoxy}}$ represent the extinction coefficients for oxyhemoglobin and deoxyhemoglobin, respectively. For simplicity, the Grüneisen parameter is assumed to be constant. Since the DW RSOM system collects signals only from depths of a few millimeters, we also assumed that there are no spectral coloring effects. Following these assumptions, the ratio *R* of the intensities recorded at 515 nm and 532 nm for the same image volume element at $\vec{{r}}$ is:

3 $$\begin{array}{ll}R\left(\vec{r}\right)=\frac{{p}_{0532}\left(\vec{r},{\lambda }_{532}\right)}{{p}_{0515}\left(\vec{r},{\lambda }_{515}\right)}=\,\frac{{\varepsilon }_{{oxy}532}{C}_{{oxy}}\left(\vec{r}\right)+{\varepsilon }_{{deoxy}532}{C}_{{deoxy}}\left(\vec{r}\right)}{{\varepsilon }_{{oxy}515}{C}_{{oxy}}\left(\vec{r}\right)+{\varepsilon }_{{deoxy}515}{C}_{{deoxy}}\left(\vec{r}\right)}\\\qquad\;=\frac{{\varepsilon }_{{oxy}532}+{\varepsilon }_{{deoxy}532}{C}_{{deoxy}}\left(\vec{r}\right)/{C}_{{oxy}}\left(\vec{r}\right)}{{\varepsilon }_{{oxy}515}+{\varepsilon }_{{deoxy}515}{C}_{{deoxy}}\left(\vec{r}\right)/{C}_{{oxy}}\left(\vec{r}\right)}\end{array}$$

Introducing Eq. 1 into Eq. 3 yields:

4 $$R\left(\vec{r}\right)=\frac{{\varepsilon }_{{oxy}532}{{sO}}_{2}\left(\vec{r}\right)+{\varepsilon }_{{deoxy}532}{(1-{sO}}_{2}\left(\vec{r}\right))}{{\varepsilon }_{{oxy}515}{{sO}}_{2}\left(\vec{r}\right)+{\varepsilon }_{{deoxy}515}{(1-{sO}}_{2}\left(\vec{r}\right))}$$

Therefore, the ratio of intensities collected at the two wavelengths is a function of sO2_._ The graph in Fig. 1c represents the ratio R as a function of oxygen saturation. Since the sO2 is a relative parameter that has values from 0 to 1, the ratio values can be estimated to range from 1.42 to 2.12. Figure 1c can be treated as a look-up table that allows obtaining an SO2 estimation from the values in two images, using Eq. 4.

### Microvessel segmentation and computation of sO2 and related parameters

In order to segment the microvascular structure within sO2 maps computed by Eq. 4, we applied a threshold to all the voxels with an sO2 value above 0.2, as exemplified in Fig. 1d. The threshold value was selected by analyzing the SNR of the images. The thresholded image reveals a complex pattern of intertwined venules and arterioles. Quantification of oxygenation changes during the vascular occlusion tests was based on computing the mean sO2 observed in all image voxels segmented, based on Eq. 4. For demonstration, microvessels were color-coded blue for sO2 values below 0.9 (representing venules) and red for sO2 values above 0.9 (representing arterioles)^33^, as depicted in Fig. 1d.

We further computed maximal hyperemic response, the ischemia downslope, the oxygenation upslope, and time to recovery by analyzing sO2 values as a function of time during the PORH test. The maximal hyperemic response was calculated as the maximum value of mean oxygen saturation obtained after the cuff was released and reported as a percentage of the lowest measured oxygen saturation value. The ischemia upslope was computed as the time needed to reach the maximal hyperemic response after release. The ischemia downslope was determined by calculating the mean slope of the decreasing part of the hyperemic curve, expressed in minutes per unit of oxygen saturation. For simplicity, the results are presented in minutes. All the values of all the parameters involved in the clinical studies are shown as average values across all subjects together with the corresponding standard deviations.

### Blood gas analysis (BGA)

BGA was performed using a commercial system (BGA, Combiline, Medizintechnik Hadler & Braun GmbH, Germany). Blood was drawn from the dermal layer in close proximity to the imaged regions.

## Data Availability

The code and raw optoacoustic imaging data of volunteers that support the findings of this study are available from the corresponding author upon request after permission is obtained from the responsible authorities and Ethics Committee of TUM University Hospital, Hospital Rechts der Isar, Technical University of Munich, Munich, Germany.

## References

[1] Roy, T. K. & Secomb, T. W. Effects of impaired microvascular flow regulation on metabolism-perfusion matching and organ function. *Microcirculation* **28**, e12673 (2021). 33236393 10.1111/micc.12673 PMC9119019

[2] Buono, M. G. D. et al. Coronary microvascular dysfunction across the spectrum of cardiovascular diseases. *JACC* **78**, 1352–1371 (2021). 34556322 10.1016/j.jacc.2021.07.042 PMC8528638

[3] Anderson, K. C., Liu, J. & Liu, Z. Interplay of fatty acids, insulin and exercise in vascular health. *Lipids Health Dis.* **24**, 4 (2025). 39773723 10.1186/s12944-024-02421-5 PMC11706162

[4] Kannenkeril, D. et al. Retinal capillary damage is already evident in patients with hypertension and prediabetes and associated with HbA1c levels in the nondiabetic range. *Diab. Care* **45**, 1472–1475 (2022). 10.2337/dc21-1569 35344581

[5] Jing, A. et al. Expression-based analyses indicate a central role for hypoxia in driving tumor plasticity through microenvironment remodeling and chromosomal instability. *npj Syst. Biol. Appl.* **4**, 38 (2018). 30374409 10.1038/s41540-018-0074-z PMC6200725

[6] Simonsen, T. G., Gaustad, J.-V. & Rofstad, E. K. Development of hypoxia in a preclinical model of tumor micrometastases. *Int. J. Radiat. Oncol. Biol. Phys.* **76**, 879–888 (2010). 20159362 10.1016/j.ijrobp.2009.09.045

[7] Knieling, F., Lee, S. & Ntziachristos, V. A primer on current status and future opportunities of clinical optoacoustic imaging. *npj Imaging* **3**, 4 (2025). 40603705 10.1038/s44303-024-00065-9 PMC12091691

[8] Organization, W. H. *Sepsis - Key Facts*. https://www.who.int/news-room/fact-sheets/detail/sepsis/ (2024).

[9] Aksu, U., Yavuz-Aksu, B. & Goswami, N. Microcirculation: current perspective in diagnostics, imaging, and clinical applications. *J. Clin. Med.* **13**, 6762 (2024). 39597906 10.3390/jcm13226762 PMC11595220

[10] Bateman, R. M., Sharpe, M. D. & Ellis, C. G. Bench-to-bedside review: microvascular dysfunction in sepsis—hemodynamics, oxygen transport, and nitric oxide. *Crit. Care* **7**, 359–373 (2003). 12974969 10.1186/cc2353 PMC270719

[11] Fink, M. Cytopathic hypoxia in sepsis. *Acta Anaesthesiol. Scand. Suppl.* **110**, 87–95 (1997). 9248546 10.1111/j.1399-6576.1997.tb05514.x

[12] Omar, M., Aguirre, J. & Ntziachristos, V. Optoacoustic mesoscopy for biomedicine. *Nat. Biomed. Eng.* **3**, 354–370 (2019). 30988470 10.1038/s41551-019-0377-4

[13] Omar, M., Soliman, D., Gateau, J. & Ntziachristos, V. Ultrawideband reflection-mode optoacoustic mesoscopy. *Opt. Lett.* **39**, 3911–3914 (2014). 24978769 10.1364/OL.39.003911

[14] Omar, M., Gateau, J. & Ntziachristos, V. Raster-scan optoacoustic mesoscopy in the 25–125 MHz range. *Opt. Lett.* **38**, 2472–2474 (2013). 23939084 10.1364/OL.38.002472

[15] Aguirre, J. et al. Precision assessment of label-free psoriasis biomarkers with ultra-broadband optoacoustic mesoscopy. *Nat. Biomed. Eng.* **1**, 0068 (2017).

[16] Schwarz, M., Garzorz-Stark, N., Eyerich, K., Aguirre, J. & Ntziachristos, V. Motion correction in optoacoustic mesoscopy. *Sci. Rep.* **7**, 10386 (2017). 28871184 10.1038/s41598-017-11277-y PMC5583247

[17] Hindelang, B. et al. Enabling precision monitoring of psoriasis treatment by optoacoustic mesoscopy. *Sci. Transl. Med.* **14**, eabm8059 (2022). 35544596 10.1126/scitranslmed.abm8059

[18] He, H. et al. Single-capillary endothelial dysfunction resolved by optoacoustic mesoscopy. *Light Sci. Appl.* **15**, 37 (2025). 10.1038/s41377-025-02103-6 PMC12764958 41484072

[19] Bezemer, R. et al. Assessment of tissue oxygen saturation during a vascular occlusion test using near-infrared spectroscopy: the role of probe spacing and measurement site studied in healthy volunteers. *Crit. Care* **13**, S4 (2009). 19951388 10.1186/cc8002 PMC2786106

[20] Haedicke, K. et al. High-resolution optoacoustic imaging of tissue responses to vascular-targeted therapies. *Nat. Biomed. Eng.* **4**, 286–297 (2020). 32165736 10.1038/s41551-020-0527-8 PMC7153756

[21] Schwarz, M., Buehler, A., Aguirre, J. & Ntziachristos, V. Three-dimensional multispectral optoacoustic mesoscopy reveals melanin and blood oxygenation in human skin in vivo. *J. Biophotonics* **9**, 55–60 (2016). 26530688 10.1002/jbio.201500247

[22] Li, X. et al. Structural and functional imaging of psoriasis for severity assessment and quantitative monitoring of treatment response using high-resolution optoacoustic imaging. *Photoacoustics* **38**, 100611 (2024). 38764522 10.1016/j.pacs.2024.100611 PMC11101711

[23] Kragelj, R., Jarm, T. & Miklavčič, D. Reproducibility of parameters of postocclusive reactive hyperemia measured by near infrared spectroscopy and transcutaneous oximetry. *Ann. Biomed. Eng.* **28**, 168–173 (2000). 10710188 10.1114/1.241

[24] Taylor-Williams, M. et al. Multispectral imaging of nailfold capillaries using light-emitting diode illumination. *J. Biomed. Opt.* **27**, 126002 (2022). 36519074 10.1117/1.JBO.27.12.126002 PMC9743620

[25] Kragelj, R., Jarm, T., Erjavec, T., Prešern-Štrukelj, M. & Miklavčič, D. Parameters of postocclusive reactive hyperemia measured by near infrared spectroscopy in patients with peripheral vascular disease and in healthy volunteers. *Ann. Biomed. Eng.* **29**, 311–320 (2001). 11339328 10.1114/1.1359451

[26] Attia, A. B. E. et al. A review of clinical photoacoustic imaging: current and future trends. *Photoacoustics* **16**, 100144 (2019). 31871888 10.1016/j.pacs.2019.100144 PMC6911900

[27] Dima, A. & Ntziachristos, V. Non-invasive carotid imaging using optoacoustic tomography. *Opt. Express* **20**, 25044–25057 (2012). 23187270 10.1364/OE.20.025044

[28] Neuschmelting, V. et al. Performance of a multispectral optoacoustic tomography (MSOT) system equipped with 2D vs. 3D handheld probes for potential clinical translation. *Photoacoustics* **4**, 1–10 (2016). 27069872 10.1016/j.pacs.2015.12.001 PMC4811917

[29] Koch, M. & Ntziachristos, V. Advancing surgical vision with fluorescence imaging. *Annu Rev. Med.* **67**, 153–164 (2016). 26768238 10.1146/annurev-med-051914-022043

[30] Pleijhuis, R. G. et al. Near-infrared fluorescence (NIRF) imaging in breast-conserving surgery: Assessing intraoperative techniques in tissue-simulating breast phantoms. *Eur. J. Surg. Oncol.* **37**, 32–39 (2011). 21106329 10.1016/j.ejso.2010.10.006

[31] Schwarz, M. et al. Optoacoustic Dermoscopy of the Human Skin: Tuning Excitation Energy for Optimal Detection Bandwidth With Fast and Deep Imaging in vivo. *IEEE Trans. Med. Imaging* **36**, 1287–1296 (2017). 28278460 10.1109/TMI.2017.2664142

[32] Aguirre, J. et al. Motion quantification and automated correction in clinical RSOM. *IEEE Trans. Med. Imaging* **38**, 1340–1346 (2019). 30676947 10.1109/TMI.2018.2883154

[33] Heitmar, R. & Safeen, S. Regional differences in oxygen saturation in retinal arterioles and venules. *Graefe’s. Arch. Clin. Exp. Ophthalmol.* **250**, 1429–1434 (2012). 22395204 10.1007/s00417-012-1980-1

